# Funeral of Professor Tommasini

**Published:** 1847-04

**Authors:** 


*Funeral of Professor Tommasini.*—The funeral of the late Prof. Tommasini is said to have taken place recently at Parma with imposing splendour. The whole of the court, and more than three hundred families of distinction, were present at the ceremony. The members of the various medical faculties, and all the dignitaries in the army and in the state, were among the followers. The leaden coffin was borne to the church of the Campo Santo by the students of the Faculty of Medicine. The funeral took place at night, and as the procession passed through the streets, the front of each house was illuminated by torches, the last testimony of the sorrowful respect and gratitude of a nation.—*Ibid, from Gaz. Med.*

